# ClonalFrameML: Efficient Inference of Recombination in Whole Bacterial Genomes

**DOI:** 10.1371/journal.pcbi.1004041

**Published:** 2015-02-12

**Authors:** Xavier Didelot, Daniel J. Wilson

**Affiliations:** 1 Department of Infectious Disease Epidemiology, Imperial College, London, United Kingdom; 2 Nuffield Department of Medicine, University of Oxford, John Radcliffe Hospital, Oxford, United Kingdom; 3 Wellcome Trust Centre for Human Genetics, Oxford, United Kingdom; UCSD, United States of America

## Abstract

Recombination is an important evolutionary force in bacteria, but it remains challenging to reconstruct the imports that occurred in the ancestry of a genomic sample. Here we present ClonalFrameML, which uses maximum likelihood inference to simultaneously detect recombination in bacterial genomes and account for it in phylogenetic reconstruction. ClonalFrameML can analyse hundreds of genomes in a matter of hours, and we demonstrate its usefulness on simulated and real datasets. We find evidence for recombination hotspots associated with mobile elements in *Clostridium difficile* ST6 and a previously undescribed 310kb chromosomal replacement in *Staphylococcus aureus* ST582. ClonalFrameML is freely available at http://clonalframeml.googlecode.com/.

## Introduction

Following recent developments in sequencing technologies, both the time and cost required to sequence whole bacterial genomes have dropped to levels where it is now being applied in clinical and public health microbiology [1,2]. On its own, the genome of a single bacterial isolate can indicate many clinically important features such as the species and strain [3,4], the level of virulence [5,6] and antimicrobial resistance properties [7,8]. Comparisons of multiple bacterial genomes can be used to investigate within-host diversity and evolution [9,10], to delineate and reconstruct local outbreaks [11–13], or to describe the global population structure and epidemiology [14,15]. Such comparisons typically involve the construction of a phylogenetic tree to reflect the relationships between genomes. One of the most popular approaches to do this is the maximum likelihood (ML) method, as implemented for example in PhyML [16], RAxML [17] and FastTree [18]. Bayesian methods such as BEAST [19] or MrBayes [20] are also frequently used, but less often than other methods because Bayesian methods tend to be more computationally expensive for applications to large genomic sets.

Phylogenetic reconstruction by any method is problematic because bacteria occasionally undergo homologous recombination, whereby a fragment of the recipient’s genome is replaced by that of the donor [21]. There are three different mechanisms that can lead to homologous recombination in bacteria: transduction where a virus transmits DNA from the donor to the recipient, transformation where donor DNA in the environment is free to be taken up by the recipient, and conjugation where donor and recipient come into direct contact [22]. The frequency of recombination varies from species to species [23], and sometimes also from one lineage to another within a single species, for example in *Clostridium difficile* [24] and *Streptococcus pneumoniae* [25]. Even in *Staphylococcus aureus* which is often described as a clonal species, some branches of the ML phylogeny contain significant evidence for recombination [26]. Ignoring recombination altogether when reconstructing a bacterial phylogeny is likely to be misleading about the true clonal relationships between isolates [27–29]. For example, the signal of temporal evolution was only detectable in *S. pneumoniae* once recombined regions had been excluded prior to phylogenetic reconstruction [30]. To correctly infer phylogenetic relationships it is therefore necessary to detect and account for recombination, but doing so also allows the study of recombination which is an interesting and important evolutionary phenomenon in its own right. For example, recombination played a key role in host adaptation of *Salmonella enterica* [31] and *Campylobacter jejuni* [32,33], in the evolution of *C. difficile* cell surface [34] and pathogenicity [6], in *Helicobacter pylori* within-host diversification [35,36] and global population structure [37,38], and in *S. pneumoniae* evolution and vaccine escape [25,39,40].

In the absence of recombination, all genomic positions would be in the clonal frame and a phylogenetic reconstruction would therefore reflect the clonal genealogy [41–43]. The ClonalFrame software [44] attempts to reconstruct this tree of clonal relationships between isolates by detecting the location of recombined regions on each branch. However, ClonalFrame was developed almost ten years ago primarily for application to multi-locus sequence typing data [45], and it is not able to deal with the large amounts of whole genome sequencing data currently being generated. For that reason, we developed a completely new implementation called ClonalFrameML which allows ML inference to be performed under the ClonalFrame model for hundreds of whole genomes in a matter of hours.

### Design and Implementation

**The ClonalFrame model of recombination**. To consider the effect of recombination on phylogenetic reconstruction, it is useful to distinguish two types of events, namely imports from a source within the population under study, and imports from an external source. The former is especially relevant if the sampled genomes cover a whole species or several species, in which case recombination typically originates from within the same species, does not introduce new polymorphism but does result in homoplasy and genetic incompatibility [46]. On the other hand, if recombination comes from an external source, then the recombined segments contain a high number of substitutions which are not seen elsewhere in the dataset [46]. This is especially relevant if the genomes under study are all from a single lineage (for example a single sequence type according to multi-locus sequence typing [45]), with frequent imports from other lineages. In this case, recombination with other members of the same lineage might also occur but would have little effect (typically none) compared to imports from other lineages because of the low diversity within the lineage.

A simple model of recombination from external sources has previously been proposed and Bayesian inference under this model is implemented in ClonalFrame [44]. In this model, genomic evolution occurs on the branches of the clonal genealogy via point mutation and recombination respectively at rates *θ*/2 and *R*/2 per site per coalescent unit of time (which is equal to the effective population size *N_e_* times the duration *g* of a generation). Note that other models use a different parameterisation involving the scaled rate of occurrence of either initiation or termination of recombination, *ρ* = 2*R* [47–49]. Recombination is assumed to affect segments of length exponentially distributed with mean *δ* in which each site is substituted with probability *ν*, irrespective of whether recombination involved transduction, transformation or conjugation.

**Overview of the ClonalFrameML algorithm**. Here we describe a new algorithm for inference under the ClonalFrame model, ClonalFrameML, which proceeds in the following steps:

1. An ML tree is constructed using standard software such as PhyML [16] or RAxML [17] and taken to be the initial clonal genealogy.
2. The ancestral sequences at internal nodes of the clonal genealogy, and any missing base calls in the observed sequences, are reconstructed by ML using a previously described algorithm [50].
3. A Baum-Welch Expectation-Maximisation (EM) algorithm is used to obtain ML estimates of the recombination parameters and the branch lengths of the clonal genealogy.
4. The ML importation status is inferred at every site using a Viterbi algorithm.
5. Uncertainty in the parameters is quantified using a bootstrapping method.

**Description of the ClonalFrame model as a hidden Markov model**. We assume the parameters *R*/*θ, δ* and *ν* are the same for all branches, and that the length of branch *i*, in terms of the expected number of mutations, is *M_i_*. Unlike ClonalFrame, which assumes a coalescent prior on the genealogy, the use of an ML tree makes no such assumption. We have found that the topology of the clonal genealogy can be estimated extremely well by ML from whole genome data [51]. Since we use an ML tree, we measure the lengths of branches and the recombination rate in units of expected numbers of mutations, whereas the ClonalFrame method measures them in units of *N_e_g* generations. For efficient computation, the ClonalFrame model can be thought of as a hidden Markov model (HMM, see, e.g. [52]) when the ancestral and descendant genomes for each branch of the clonal genealogy have been observed or reconstructed. The hidden state of the HMM records whether each nucleotide was subject to recombination or not on the branch connecting the two genomes. Nucleotides unaffected by recombination are said to be *unimported* (*U*) and nucleotides subject to recombination are said to be *imported* (*I*) [44]. Based on the ClonalFrame model, we define the following transition probability matrix for the hidden variable between sites, *H_j_* and *H_k_* distance *d_jk_* apart:

$$\mathrm{Pr}\left(H_{k}|H_{j}\right)=\left\{\begin{array}{cccc} e^{-d_{jk}M\frac{R}{\theta }} & & H_{j}=U\;\mathrm{and}\;H_{k}=U & \\ 1-e^{-d_{jk}M\frac{R}{\theta }} & & H_{j}=U\;\mathrm{and}\;H_{k}=I & \\ 1-e^{-d_{jk}/\delta } & & H_{j}=I\;\mathrm{and}\;H_{k}=U & \\ e^{-d_{jk}/\delta } & & H_{j}=I\;\mathrm{and}\;H_{k}=I & \end{array}\right.$$

Again following the ClonalFrame model, we define the following emission probabilities for the data at nucleotide *j*, which define the likelihood for the ancestral and descendant sequences conditional on the underlying hidden variable:

$$\mathrm{Pr}(A_{j},D_{j}|H_{j})=\left\{\begin{array}{cc} p_{A_{j}D_{j}}^{\left(M\right)} & H_{j}=U\\ p_{A_{j}D_{j}}^{\left(\nu \right)} & H_{j}=I \end{array}\right..$$

Here *A_j_* and *D_j_* are the nucleotides of the ancestral and descendant sequences and $p_{jk}^{\left(t\right)}$ is the transition probability from nucleotide *j* to *k* in time *t* under the HKY85 model [53].

**ML inference under the ClonalFrame model**

In the EM algorithm that follows, we approximate the transition and emission probabilities to obtain analytic results for the parameter updates. We employ a Poisson approximation to the transition probabilities that, in effect, assumes no more than a single transition between adjacent sites:

$$\mathrm{Pr}(H_{k}|H_{j})\approx \left\{\begin{array}{cccc} e^{-d_{jk}M\frac{R}{\theta }} & & H_{j}=U\;\mathrm{and}\;H_{k}=U & \\ \left(d_{jk}M\frac{R}{\theta }\right)e^{-d_{jk}M\frac{R}{\theta }} & & H_{j}=U\;\mathrm{and}\;H_{k}=I & \\ \left(d_{jk}/\delta \right)e^{-d_{jk}/\delta } & & H_{j}=I\;\mathrm{and}\;H_{k}=U & \\ e^{-d_{jk}/\delta } & & H_{j}=I\;\mathrm{and}\;H_{k}=I & \end{array}\right.$$

We summarize the sixteen possible combinations of ancestral and descendant nucleotides by a single observation *O_j_* that records whether they are the same (*S*) or different (*D*), and employing a Poisson approximation that, in effect, assumes no more than a single substitution along the branch, then:

$$\mathrm{Pr}(A_{j},D_{j}|H_{j})\approx \left\{\begin{array}{cccc} e^{-M} & & O_{j}=S\;\mathrm{and}\;H_{j}=U & \\ M\;e^{-M} & & O_{j}=D\;\mathrm{and}\;H_{j}=U & \\ e^{-\nu } & & O_{j}=S\;\mathrm{and}\;H_{j}=I & \\ \nu \;e^{-\nu } & & O_{j}=D\;\mathrm{and}\;H_{j}=I & \end{array}\right.$$

We use a Baum-Welch EM algorithm to estimate the model parameters. Given initial parameters $\Theta =\{\frac{R}{\theta },\delta ,\nu ,M_{1\ldots B}\}$ comprising the recombination parameters and the *B* branch lengths, we use the forward-backward algorithm to calculate the expected number of transitions, *T_ijk_* between the hidden states *j* and *k* for sites less than 1kb apart, and the expected number of observations, *E_ijk_*, of state *k* given hidden state *j* on branch *i*. The 1kb restriction helps ensure the validity of the Poisson approximation. We then update the parameters as follows

$${M^{\prime }}_{i}=\frac{\alpha_{M}+E_{iUD}}{\beta_{M}+E_{iUS}+E_{iUD}}$$,

$$\nu^{\prime }=\frac{\alpha_{\nu }+\sum_{i=1}^{B}E_{iID}}{\beta_{\nu }+\sum_{i=1}^{B}E_{iIS}+E_{iID}}$$,

$$\frac{1}{\delta^{\prime }}=\frac{\alpha_{\delta }+\sum_{i=1}^{B}T_{iIU}}{\beta_{\delta }+\bar{d}\sum_{i=1}^{B}(T_{iIU}+T_{iII})}$$,

$${\left(\frac{R}{\theta }\right)}^{\prime }=\frac{\alpha_{\frac{R}{\theta }}+\sum_{i=1}^{B}T_{iUI}}{\beta_{\frac{R}{\theta }}+\bar{d}\sum_{i=1}^{B}M_{i}\left(T_{iUU}+T_{iUI}\right)}$$,

where *α* and *β* represent prior information in the form of pseudocounts for the various parameters and $\overset{‒}{d}$ is the mean distance between adjacent called sites less than 1kb apart. The prior information conveyed by the pseudocounts is analogous to a gamma prior distribution with shape and rate parameters *α* and *β*. In the analyses presented in this paper we set the prior means, *α*/*β*, equal to *M* = 10^−4^, ν = 10^−1^ 1/δ = 10^−3^, and *R/θ* = 10^−1^, and the prior standard deviations, $\sqrt{\alpha }/\beta$, equal to the prior means, representing prior uncertainty over roughly three orders of magnitude.

**Quantifying the uncertainty in the parameters**. To obtain a measure of uncertainty in the parameter estimates, we perform a parametric bootstrap where we simulate the number of transitions *T_ijk_* and emissions *E_ijk_* of each sort based on the ML parameter estimates using a posterior decoding algorithm [52]. The parameters are then drawn from gamma distributions with shape and rate parameters given by the numerators and denominators respectively in the above equations. This computationally efficient but somewhat heuristic procedure accounts for uncertainty in *T_ijk_* and *E_ijk_*, and for uncertainty in the parameters given *T_ijk_* and *E_ijk_*, but not in the tree topology or ancestral state reconstruction, so it will underestimate the true uncertainty in the parameter estimates.

**Extended model with separate recombination parameters for each branch**. To allow detection of heterogeneity in the recombination process on different branches of the tree, we implemented an EM algorithm that estimates parameters for each branch, using a hyperprior in the form of pseudocounts to help obtain sensible values for uninformative branches. Formally, we defined the per-branch recombination parameters to be related to the mean recombination parameters through a branch-specific factor as follows:

$$\lambda_{i}=\bar{\lambda }w_{i}^{\left(\lambda \right)}$$

where *λ* represents one of the parameters, $\bar{\lambda }$ is the mean of that parameter and *w_i_* is the branch-specific factor. This leads to the following EM updates. First, the mean branch length parameter is iteratively updated until the following equation converges:

$${\bar{M}}^{\prime }=\frac{\alpha_{M}+\sum_{i=1}^{B}E_{iUD}}{\beta_{M}+\sum_{i=1}^{B}\left(\alpha_{w}+E_{iUD}\right)\left(E_{iUS}+E_{iUD}\right)/\left(\beta_{w}+\bar{M}\left(E_{iUS}+E_{iUD}\right)\right)}$$

Then the individual branch length factors are updated as follows:

$${w^{\prime }}_{i}^{\left(M\right)}=\left(\alpha_{w}+E_{iUD}\right)/\left(\beta_{w}+{\bar{M}}^{\prime }\left(E_{iUS}+E_{iUD}\right)\right)$$

The updating equations are similar for the other parameters:

$${\bar{\nu }}^{\prime }=\frac{\alpha_{\nu }+\sum_{i=1}^{B}E_{iID}}{\beta_{\nu }+\sum_{i=1}^{B}\left(\alpha_{w}+E_{iID}\right)\left(E_{iIS}+E_{iID}\right)/\left(\beta_{w}+\bar{\nu }\left(E_{iIS}+E_{iID}\right)\right)}$$,

$${w^{\prime }}_{i}^{\left(\nu \right)}=\left(\alpha_{w}+E_{iID}\right)/\left(\beta_{w}+{\bar{\nu }}^{\prime }\left(E_{iIS}+E_{iID}\right)\right)$$,

$$\frac{1}{{\bar{\delta }}^{\prime }}=\frac{\alpha_{\delta }+\sum_{i=1}^{B}T_{iIU}}{\beta_{\delta }+\sum_{i=1}^{B}\left(\alpha_{w}+T_{iIU}\right)\bar{d}\left(T_{iIU}+T_{iII}\right)/\left(\beta_{w}+\bar{d}\left(T_{iIU}+T_{iII}\right)/\bar{\delta }\right)}$$,

$${w^{\prime }}_{i}^{\left(\delta \right)}=\left(\alpha_{w}+T_{iIU}\right)/\left(\beta_{w}+\bar{d}\left(T_{iIU}+T_{iII}\right)/{\bar{\delta }}^{\prime }\right)$$,

$${\bar{\left(\frac{R}{\theta }\right)}}^{\prime }=\frac{\alpha_{\frac{R}{\theta }}+\sum_{i=1}^{B}T_{iUI}}{\beta_{\frac{R}{\theta }}+\sum_{i=1}^{B}\left(\alpha_{w}+T_{iUU}\right)M_{i}\bar{d}\left(T_{iUU}+T_{iUI}\right)/\left(\beta_{w}+\bar{\left(\frac{R}{\theta }\right)}M_{i}\bar{d}\left(T_{iUU}+T_{iUI}\right)\right)}$$,

$${w^{\prime }}_{i}^{\left(\frac{R}{\theta }\right)}=\left(\alpha_{w}+T_{iUU}\right)/\left(\beta_{w}+{\bar{\left(\frac{R}{\theta }\right)}}^{\prime }M_{i}\bar{d}\left(T_{iUU}+T_{iUI}\right)\right)$$.

To ensure $\bar{\lambda }$ was the mean of parameter *λ* across branches we set *α_w_* = *β_w_* and we chose an absolute value of 100 to prevent poorly identified parameters from deviating far from the mean.

## Results

### Example application to a simulated dataset

**Figure 1:**
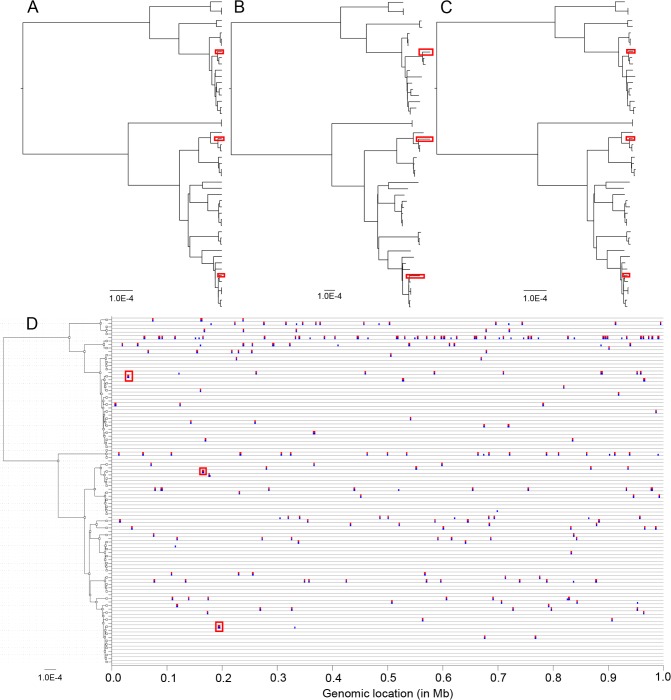
An example application of ClonalFrameML to a simulated dataset. (A) The clonal genealogy produced by simulation. (B) Maximum-likelihood reconstructed phylogeny. (C) ClonalFrameML reconstructed phylogeny. (D) Representation of recombination events along the genome for each branch of the genealogy in (A). True events are shown in blue and events detected by ClonalFrameML are shown in red. Three branches of interest and their associated recombination events are highlighted by red boxes.

To illustrate the working of ClonalFrameML, we simulated under the ClonalFrame model [44] a dataset made of 50 genomes of 1Mb each. The clonal genealogy was taken from the coalescent model [54] with a scaled mutation rate of *θ* = 10^−3^ per site (Fig. 1A). The ratio of recombination and mutation rates, the mean length of imports and the average distance of the imports were equal to *R*/*θ* = 0.0626, *δ* = 554.95 bp and *ν* = 0.0374, respectively. The first step of our inference procedure is to compute an ML phylogeny, and here PhyML [16] took approximately one minute on a standard desktop computer to produce the ML tree (Fig. 1B). This tree has the same topology as the true clonal genealogy (Fig. 1A). This is because in the ClonalFrame model, recombination has an external origin so that the substitutions imported on a given branch are shared by the genomes that descend from that branch. Recombination is therefore informative about the tree topology in exactly the same way as mutation, which is why the tree topology reconstructed by the ML phylogeny is correct even when no attempt is made to account for recombination.

The scale of branch lengths in the reconstructed phylogeny (Fig. 1B) was 2.1 times greater than in the true tree (Fig. 1A), because the latter accounts only for the substitutions introduced by mutation whereas the former also includes the differences imported by recombination. The relative effect of recombination and mutation [55] was equal to *r*/*m* = (*R*/*θ*) × *δ* × *ν* = 1.3 so that recombination introduced a similar number of substitutions as did mutation, which explains the difference in the reconstructed scale. Apart from this important difference in the scale, the relative lengths of branches in the reconstructed phylogeny were approximately consistent with the correct genealogy. This is because the substitutions introduced by recombination accumulate in the genomes in a clock-like manner, as do mutations. The most noticeable difference concerned some of the shortest terminal branches in the true clonal genealogy, which had lengths several times longer in the reconstructed phylogeny (see examples of this marked by red boxes in Fig. 1B). The overestimation of these terminal branch lengths could have important consequences, for example it could mislead one into excluding the possibility of direct transmission between two infected individuals in a genomic epidemiology study [11,56–58].

ClonalFrameML was then applied to this simulated dataset using the ML phylogeny (Fig. 1B) as tree input, which took approximately 15 minutes to run on a standard desktop computer. The parameter estimates and 95% confidence intervals were as follows: *R*/*θ* = 0.061 [0.053–0.067], *δ* = 511.59bp [456.96–575.77] and *ν* = 0.0386 [0.0373–0.0397]. These three intervals included the correct values used when simulating the data. ClonalFrameML also estimates a phylogeny with corrected branch lengths (Fig. 1C). Unlike the ML tree (Fig. 1B), the scale of the branch lengths in the ClonalFrameML tree is the same as in the true phylogeny (Fig. 1A). Where short terminal branch lengths had been overestimated by the ML tree in the example data, these were corrected by ClonalFrameML (red boxes in Fig. 1C). One way to assess and compare the correctness of phylogenetic reconstructions is through a distance measure between trees known as the branch score [59]. Between the true tree (Fig. 1A) and the ML reconstruction (Fig. 1B), the branch score was equal to 1.29×10^−3^ whereas between the true tree (Fig. 1A) and the ClonalFrameML reconstruction (Fig. 1C), the branch score was lower, equal to 7.88×10^−5^. This large improvement is partly, but not exclusively, due to the difference in scales between the trees. All three trees were rescaled to have a sum of branch lengths equal to one, and the two branch scores were then equal to 0.053 and 0.019, respectively. The true and inferred recombination events were compared for all branches of the clonal genealogy (Fig. 1D). All the exemplar terminal branches that were too long in the ML phylogeny corresponded to recombination events that have been accurately detected by ClonalFrameML (red boxes in Fig. 1). There were 248 real recombination events throughout the tree, and 213 (86%) of them were correctly detected. The 35 events that were not detected tended to be short and to contain relatively few substitutions. All the detected recombination events corresponded to genuine events.

The original Monte-Carlo Markov Chain (MCMC) algorithm implemented in the ClonalFrame software [44] was applied to the same simulated dataset for comparison with ClonalFrameML. Each iteration of the MCMC took about 7.5 seconds. ClonalFrame was run for 20,000 iterations which took about 42 hours—more than a hundred times the time it took to run ClonalFrameML. The first half of the iterations were discarded as MCMC burn-in, and the second half were recorded every ten iterations to produce a sample of size 1000 from the posterior. Assessing MCMC convergence and mixing properties is always challenging, and the generally recommended method is to compare separate runs. Four separate runs were performed and found to yield comparable samples of the parameters (S1 Fig.). The four runs were combined to produce the following parameter estimates and 95% credibility intervals: *R*/*θ* = 0.056 [0.049–0.064], *δ* = 529.62 [464.47–603.50] and *ν* = 0.0386 [0.0375–0.0398]. These estimates are in good agreement with both the correct values and the estimates from ClonalFrameML. The intervals of uncertainty, often seen as one of the great advantages of fully Bayesian methods, are also similar to the ones estimated using ClonalFrameML thus suggesting that our bootstrapping method is appropriate.

### Evaluation of performance

The simulation and inference steps described above were repeated one hundred times to study the performance of ClonalFrameML under various conditions. Each simulation used different parameter values drawn uniformly on a log_10_ scale for *R*/*θ* between 0.01 and 10, for *δ* between 100 and 10,000bp and for *ν* between 0.01 and 0.1. The average running time of ClonalFrameML was 15 minutes on a standard desktop computer, with all runs taking less than an hour.

**Figure 2:**
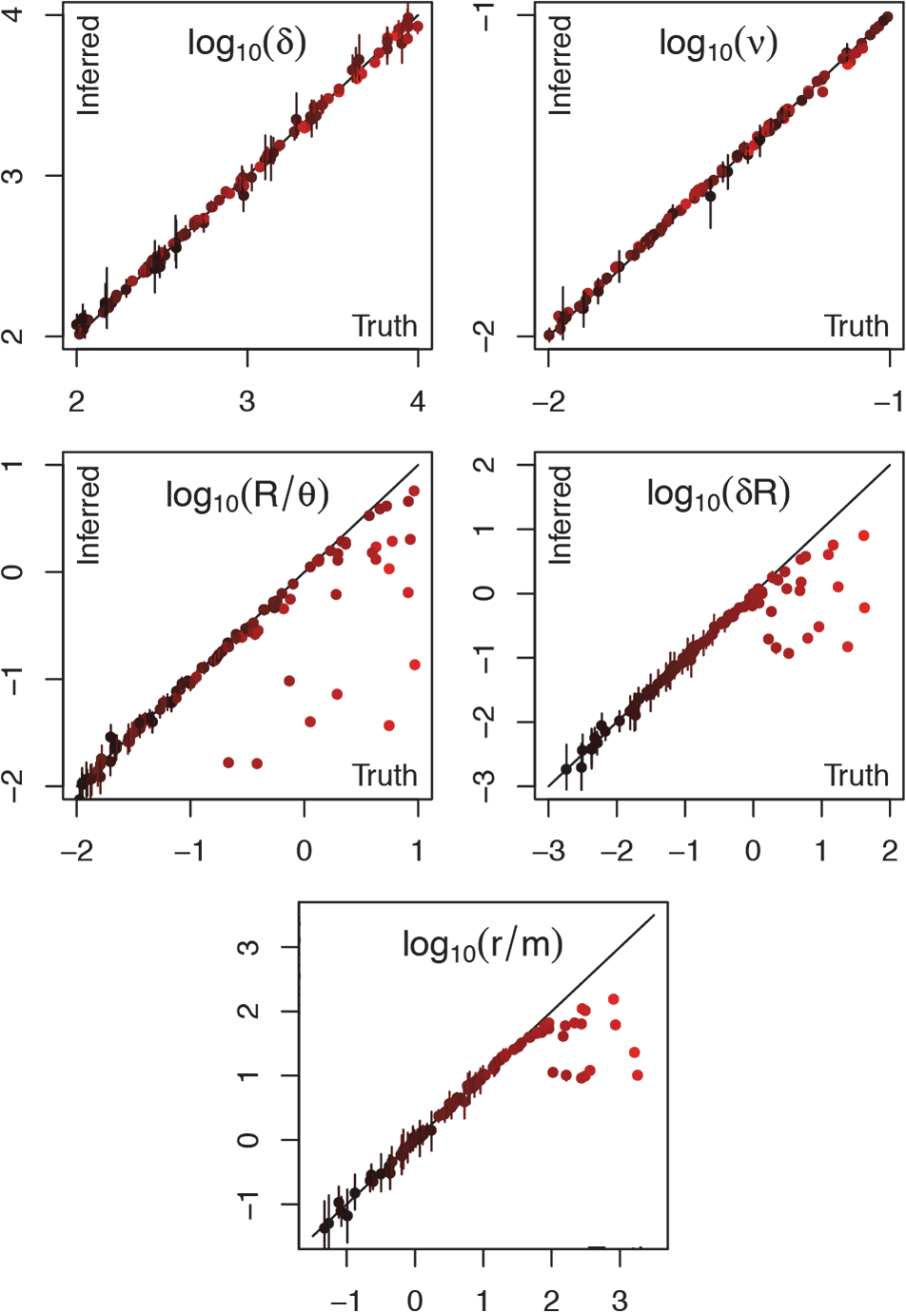
Comparison of correct parameter values with estimates from ClonalFrameML for a hundred datasets simulated under the ClonalFrame model. Dots represent the point estimates and bars the 95% confidence intervals. Colours represent the correct value of the compound parameter *δR* ranging from 10^−3^ (black) to 10^2^ (red).

The true and inferred values were compared for the parameters *R*/*θ, δ* and *ν* in each simulation (Fig. 2). The key determinant for how well the algorithm performs is the compound parameter *δR* (Fig. 2). When this parameter is below one, the inferred values are well aligned with the correct values, and the 95% confidence intervals contain the correct values in 82%, 85% and 74% of the simulations for *R*/*θ, δ* and *ν*, respectively. When *δR* is greater than one, the estimates of *δ* and *ν* remain in good alignment with the correct values, but the relative rate of recombination *R*/*θ* is sometimes underestimated. The compound parameter *δR* represents the rate at which a given site is affected by recombination on a branch of the clonal genealogy measured in coalescent units of time. When *δR* is greater than one, there is a significant chance that recombination happened more than once at any genomic position for the longer branches of the phylogeny, but this is not accounted for in the ClonalFrame model which considers that each position is either imported or not. For example, one of the simulations used *δ* = 958bp and *R* = 0.002 and the values inferred by ClonalFrameML were *δ* = 866bp and *R* = 0.0006, so that *R* was underestimated by a factor of three. In spite of this, the location of inferred recombined regions was correct but saturated for some of the long branches (S2 Fig.). Because of this saturation effect, *r*/*m* was correctly estimated as long as the correct value was below 100, but was often underestimated beyond that (Fig. 2). ClonalFrameML may therefore underestimate the recombination rate in situations where there has been so much recombination that it happened several times over for some branches. A good indication of this is provided by the presence of branches on which the whole genome has been found to be recombinant (S2 Fig.). This is a limitation of the original ClonalFrame model [44] rather than of the ML implementation presented here, but this has not been found to be a significant problem in practice, even in application to the highly recombinant *Helicobacter pylori* [60]. However, in such promiscuous species the signal of clonal inheritance is rapidly lost so that models of pure admixture may be more appropriate, such as the Structure and FineStructure models where linkage disequilibrium is caused only by linkage along the genome [37,38].

The branch score [59] was calculated to compare both the ML trees and the ClonalFrameML trees against the correct tree used in each simulation where *δR* was below one. We found that the ClonalFrameML trees were closer to the true trees than the uncorrected ML trees in all remaining simulations. The average branch score between the true and uncorrected ML trees was 7.47×10^−3^ whereas it was 9.72×10^−5^ between the true and ClonalFrameML trees (for full comparative results see S3 Fig.). A large part of this improvement is due to the fact that the overall scale of the ClonalFrameML tree is more accurate than that of the ML tree, as noted earlier. We repeated the comparison after normalizing all trees to have a sum of branch lengths equal to one. The average branch score between the true and ML trees was then 4.81×10^−2^ compared with 1.68×10^−2^ between the true and ClonalFrameML trees. There was therefore a clear improvement in the estimated branch lengths beyond the correction in scale.

### Application to simulated data with intra-population recombination

The ClonalFrame model considers that recombination events have an external source, so that they introduce substitution at a relatively high rate denoted *ν* [44]. If the dataset contained genomes covering the diversity of a whole bacterial species, there might be a few recombination events coming from other closely related species, but most events will have donors from the species under study, so that the main source of recombination is not external. This situation is best modelled by the coalescent with gene conversion [61], but drawing inference under the resulting ancestral recombination graph is a notoriously complex statistical problem [46,62,63]. Instead, here we consider the application of the ClonalFrame model of external recombination to analyse data simulated with within-population recombination.

Simulation of sequence data under the coalescent with gene conversion is implemented in the software SimMLST [64], which was used to simulate a hundred different datasets, each consisting of 50 genomes of length 1Mb. Each simulation used a mutation rate of *θ* = 10^−3^ per site, a ratio of recombination to mutation rate *R*/*θ* sampled between 0.01 and 1, and an average length of recombined fragment *δ* sampled between 100 and 10,000bp, with these two samples being taken uniformly on a log_10_ scale.

**Figure 3:**
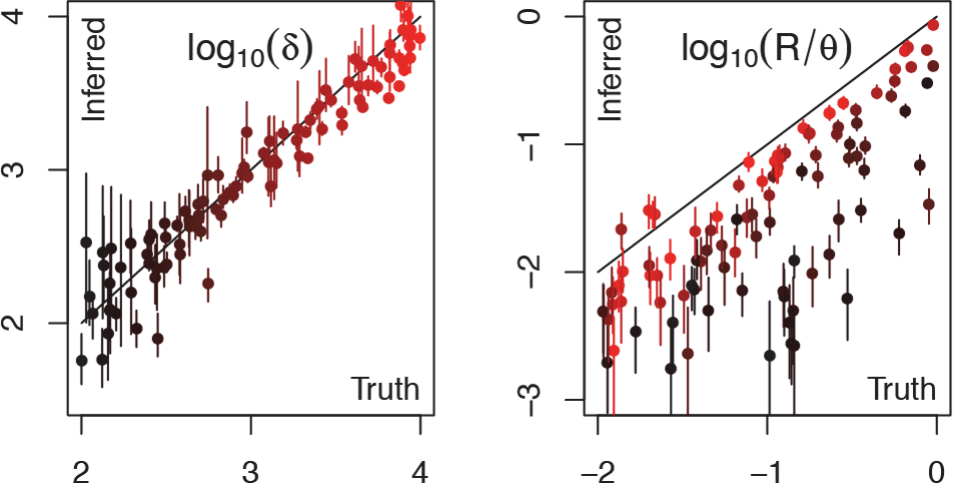
Comparison of correct parameter values with estimates from ClonalFrameML for a hundred datasets simulated under the coalescent with gene conversion model of intra-population recombination. Dots represent the point estimates and bars the 95% confidence intervals. Colours represent the correct value of the parameter δ ranging from 10^2^ (black) to 10^4^ (red).

The correct and inferred values of the two parameters *R*/*θ* and *δ* were compared for each simulation (Fig. 3). The estimates of *δ* were unbiased and strongly reflected the correct values used in simulation. The relative recombination rate *R*/*θ* was also correlated with the correct values, but almost always underestimated. This bias was especially important when the recombination tract length *δ* was short, which typically resulted in an underestimation of *R*/*θ* by an order of magnitude. For longer values of *δ* on the other hand, the bias was smaller. In datasets where the bias was important, a good indication of this was provided by large confidence intervals around both estimates of *R*/*θ* and *δ* (Fig. 3). These performance characteristics fit with our expectations given the differences between the models used for simulation and inference. Since the simulated recombination events come from within the population of interest, they introduce fewer substitutions than if they had come from an external source, which makes them more difficult to detect. When these events are relatively short, they are likely to introduce very little polymorphism if any, so that a large fraction of them becomes impossible to detect. In these conditions, there is much uncertainty about the relative rate of recombination *R*/*θ*. When the events are longer they are easier to detect, but a fraction of them will still be impossible to detect, for example if their donor was a close relative of the recipient, leading to underestimation of *R*/*θ*.

### Application to *Clostridium difficile* ST6

Detection of transmission relies on the identification of closely related bacteria. Recombination events have the potential to mask recent transmission by inflating the genomic divergence between closely related bacteria. In previous work the genomes of bacteria sampled from all cases of *Clostridium difficile* infection reported in Oxfordshire between 2007 and 2011 were sequenced to investigate the importance of transmission within hospitals [24,65]. To investigate the ability of ClonalFrameML to detect recombination in a transmission setting, we analysed 86 of these genomes sharing the same multi-locus sequence type, ST6. As previously described [24,65], these genomes were mapped to the complete sequence of reference genome CD630 [66]. However, ClonalFrameML can be equally well applied to a whole-genome alignment obtained from a de novo assembly approach. In a gene-by-gene approach, for example [67], one can scaffold local alignments using a reference genome to obtain a whole-genome alignment. ClonalFrameML took approximately 2 hours to run on our dataset. The average length of recombined fragments was estimated to be *δ* = 591bp [528–691] and the average divergence between donor and recipient was *ν* = 0.032 [0.031–0.033]. The ratio of rates of recombination and mutation was *R*/*θ* = 0.30 [0.25–0.35], whereas the ratio of effects of recombination and mutation was *r/m* = 5.67 [4.62–7.18]. This indicated that recombination happened three times less often than mutation, but because each recombination event introduced on average *δν* = 19 substitutions, recombination overall caused six times more substitutions than mutation, confirming the importance of recombination even in these closely related bacteria.

**Figure 4:**
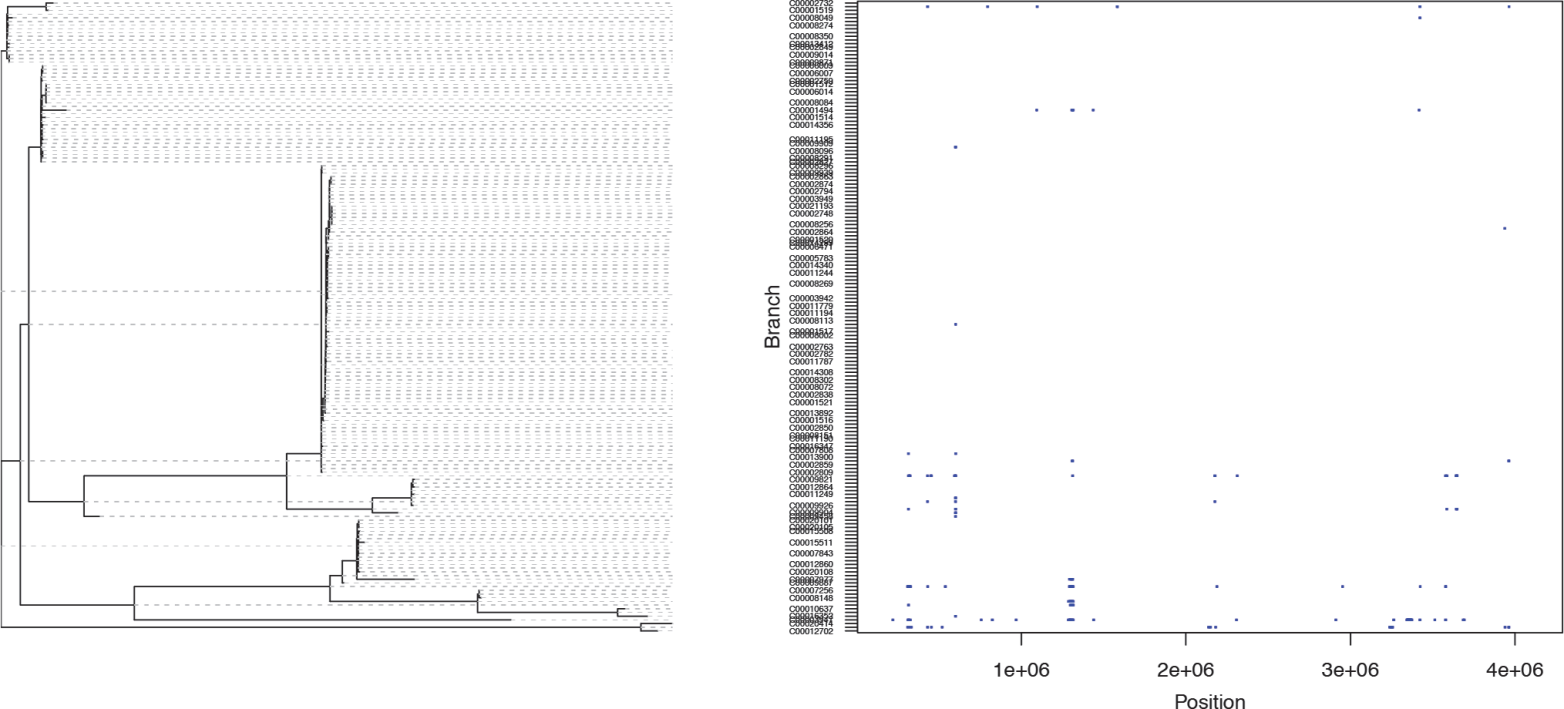
Application of ClonalFrameML to 86 genomes of *C. difficile* ST6. For any branch of the genealogy and any position along the genome, inferred recombination is marked in blue.

ClonalFrameML identified 167 recombination events on all branches of the clonal genealogy (Fig. 4). Three regions appeared to be possible hotspots of recombination, since we found multiple imports on several branches (up to seven) which would not be expected to happen by chance if events were uniformly distributed along the genome for each branch. The first hotspot spanned from 314kb to 315kb in CD630 and corresponded to the annotated *fliI* gene [66]. This gene been described as one of only two integration sites for prophage ΦCD27 [68]. The second hotspot, from position 600kb to 602kb, corresponded to the *tetM* gene which encodes a conjugative transposon tetracycline resistance protein [69]. The third hotspot, from position 1,307kb to 1313kb, contained several conjugal transfer proteins [66]. Recombination hotspots in *C. difficile* seem therefore to be caused by the presence of genomic mobile elements, as previously reported for example in *S. pneumoniae* [30] and *S. aureus* [26].

Based on a measure of the within-host mutation rate, it was previously estimated that the divergence between two *C. difficile* genomes sampled from the donor and recipient of a direct transmission event would be at most two single nucleotide polymorphisms (SNPs) [65]. We compared the distance between all pairs of genomes in the ML tree and in the corrected ClonalFrameML tree (S4 Fig.). Amongst the 86 genomes of ST6, there were 12 pairs for which the distance was greater than 2 SNPs in the ML tree and lower than 2 SNPs once recombination had been accounted for in the ClonalFrameML tree (S4 Fig.), suggesting that they may represent cases of direct transmission. This result illustrates the importance of accounting for recombination when performing genomic epidemiology investigations.

### Application to *Staphylococcus aureus*

Recombination is an important force in bacterial evolution and has played a role in shaping the population structure of many species [22,70], including those such as *Staphylococcus aureus* that have otherwise been characterized as evolving clonally [26]. Although there is limited signal of recombination within closely related lineages [15,71–75], analysis of species-wide diversity reveals evidence of widespread homoplasy in the genome [26]. Further, recombination has been shown to have played an important role in the emergence of certain lineages, notably the hospital-associated ST 34 and the globally distributed MRSA ST 239 [76,77]. STs 34 and 239 are hybrids resulting from large chromosomal replacement events. ST 34 is thought to have evolved from an ST 30 lineage via the introduction of a 244kb region from an ST 10 donor lineage [76]. ST 239 appears to have arisen from the integration of a 635kb region from an ST 30 donor into an ST 8 background [77]. In both hybrid lineages, the chromosomal replacements span the origin-of-replication.

**Figure 5:**
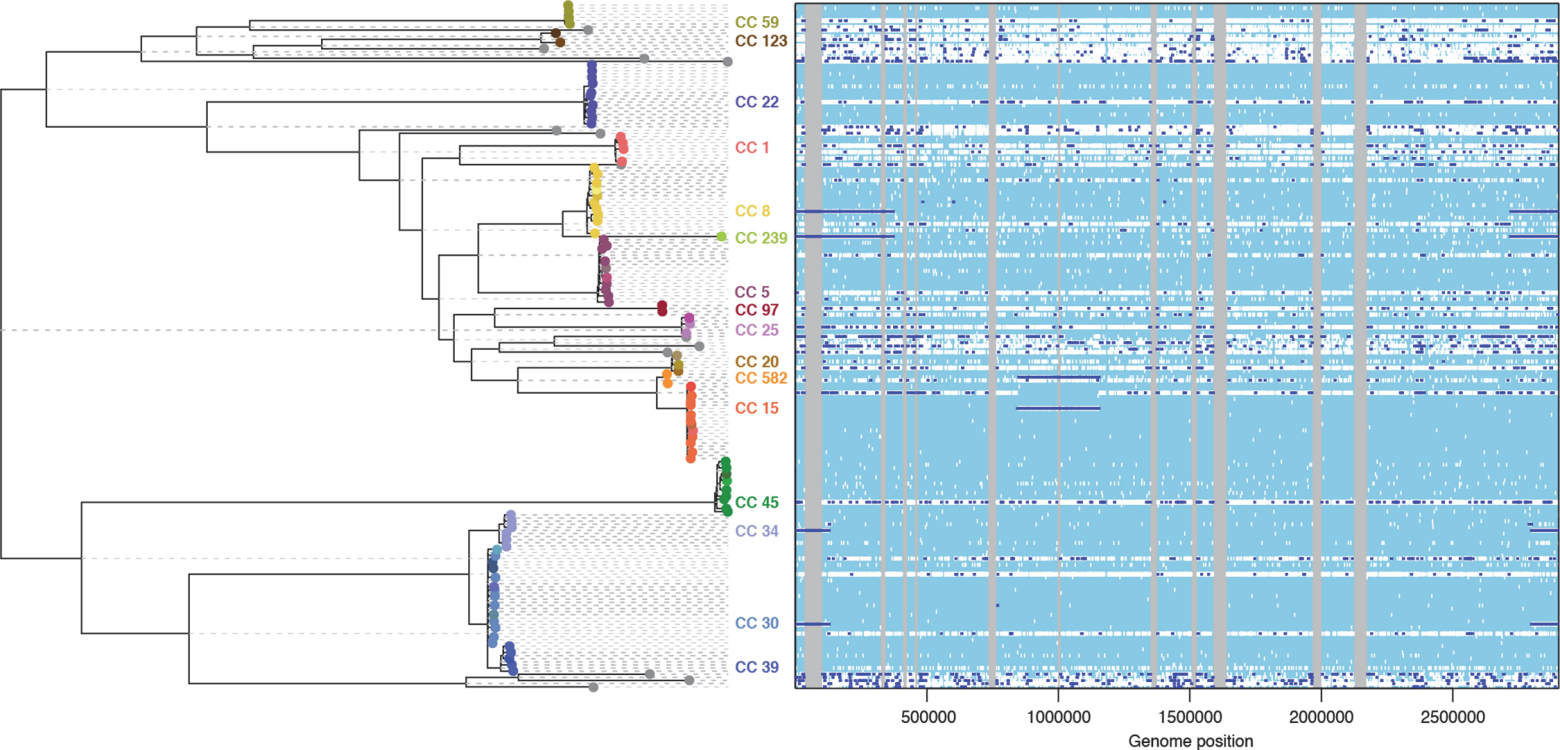
ClonalFrameML analysis of recombination in *S. aureus* based on 110 genomes representing carriage and reference isolates mapped to MRSA252. Reconstructed substitutions (white vertical bars) are shown for each branch of the ML tree. Grey areas represent non-core regions of the MRSA252 genome. Dark blue horizontal bars indicate recombination events detected by the analysis.

We applied ClonalFrameML to investigate 110 *S. aureus* carriage and reference genomes that represent species-wide diversity [26] using an extension to the standard ClonalFrame model that allows different recombination parameters to be inferred on different branches of the clonal genealogy. The mean parameters were estimated to be *R*/*θ* = 0.215, *δ* = 183bp and *ν* = 7.20×10^−3^, but substantial variation was detected between the branches of the tree (Fig. 5). In particular, large importations of 231kb and 555kb were detected, corresponding to the chromosomal replacements spanning the origin-of-replication in STs 34 and 239 respectively. Note that the positions and lengths of recombination events reported here are measured relative to the MRSA252 reference genome. Additionally, we found a new chromosomal replacement event of 310kb associated with ST 582, a close relative of ST 15. Unlike the previously described events, this large chromosomal replacement spans 845–1155kb, a region approximately 1Mb from the origin-of-replication. The nature and origin of this novel chromosomal replacement requires further investigation.

The ClonalFrameML analysis of recombination in *S. aureus* reveals a curious property of the method that we expect applies to phylogenetic methods in general. The effect is visible most clearly in the three large chromosomal replacement events ancestral to STs 34, 239 and 582. In each case, the large recombination event, marked by a dark blue horizontal line, is mirrored on the branch leading to the sister clade. This mirroring can be explained by substitution events that occurred on the branch immediately ancestral to the two sister clades. When recombination introduces DNA from a relative that does not possess these derived substitutions, then it becomes more parsimonious to attribute the mirrored substitutions to the sister clade rather than have them arise on the parent branch only to immediately revert them in the branch that receives the recombination event (S5 Fig.). This effect is likely to contribute to the well-recognized distortion of branch lengths leading to spurious inference of demography, selection and molecular clocks when phylogenetic methods are applied to recombining populations [27,28,78,79].

### Conclusion

The advent of rapid, inexpensive whole genome sequencing is revealing more than ever the importance of recombination to bacteria. Accounting for recombination in phylogenetic analyses remains a fundamental yet challenging problem, and one that has become more difficult, not easier, with the volume of information provided by hundreds or thousands of bacterial whole genomes. We have introduced a new maximum likelihood method, ClonalFrameML, that implements the model underlying the popular Bayesian ClonalFrame approach in a computationally efficient manner, and we have demonstrated its ability to estimate recombination parameters and detect importation events in the context of understanding short-term transmission dynamics and long-term bacterial evolution.

### Availability and Future Directions

ClonalFrameML is freely available from http://clonalframeml.googlecode.com/. Further work is planned to improve the front end, and to provide compatibility with the input files of the ClonalFrame software.

## Supporting Information

S1 Fig Comparison of four runs of ClonalFrame on the first simulated dataset. (PDF)

S2 Fig Example application of ClonalFrameML to a simulated dataset resulting in an underestimation of the recombination rate. True recombination events are shown in blue and events detected by ClonalFrameML are shown in red.
(PDF)

S3 Fig Comparison of branch scores for a hundred datasets simulated under the ClonalFrame model. The x-axis shows the branch score between true and ML tree, whereas the y-axis shows the branch score between true and ClonalFrameML tree. In the right panel all trees have been normalized to have a sum of branch lengths equal to one.
(PDF)

S4 Fig Comparison of the distances between pairs of genomes in the maximum likelihood tree and in the ClonalFrameML tree for the *C. difficile* application. (PDF)

S5 Fig Recombination causes homoplasy and mirroring in phylogenetic trees. The branches of a simplified tree representing *S. aureus* STs 15, 582, and 20 together with an outgroup representing all other lineages are labelled A-E. A recombination event from ST 20 to ST 582 is labelled R. Below, patterns of genetic diversity are represented for mutations arising on branches A-E in the absence of recombination (clonal sites). Recombined sites show the effect of the recombination event R on patterns of diversity. Mutation events occurring on branch D are imported into lineage A, leading to homoplasy. Mutation events that occurred on branch B are displaced by the recombination event, leading to a spurious pattern resembling mutation on branch E, which we refer to as mirroring.
(PDF)

S1 Text Instructions for installing the software and analysing the example dataset. (PDF)

S1 Table Table of mathematical notation. (DOCX)
