# Supplementary figures and images for: ClonalFrameML: Efficient Inference of Recombination in Whole Bacterial Genomes
Source: PLoS Comput Biol. 2015 Feb 12;11(2):e1004041. doi: 10.1371/journal.pcbi.1004041 (PMC4326465; doi:10.1371/journal.pcbi.1004041)

$R/\theta$  $\delta$  $v$  $r/m$ 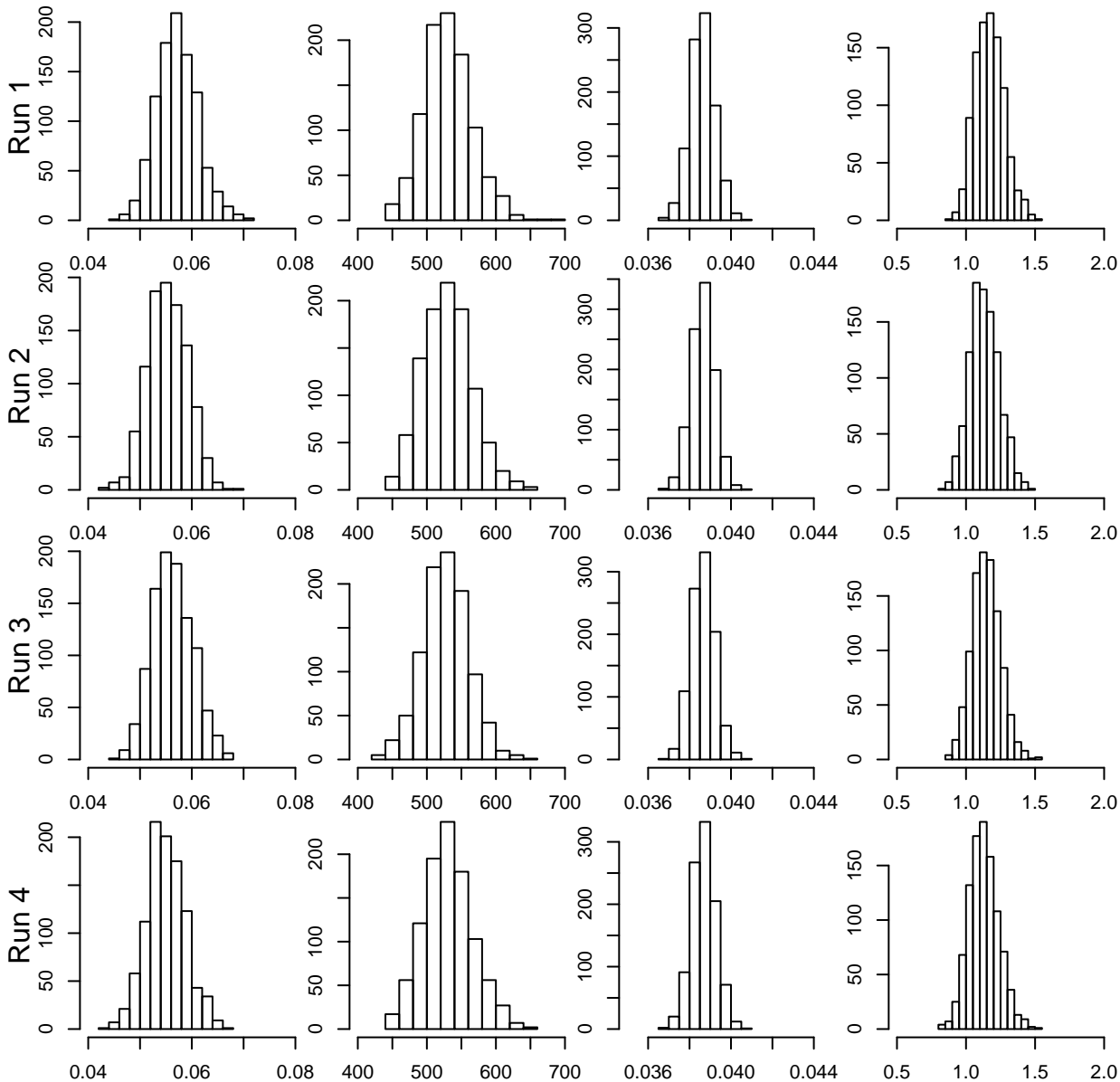

Supplement: S1 Fig — (PDF) [file pcbi.1004041.s001.pdf]

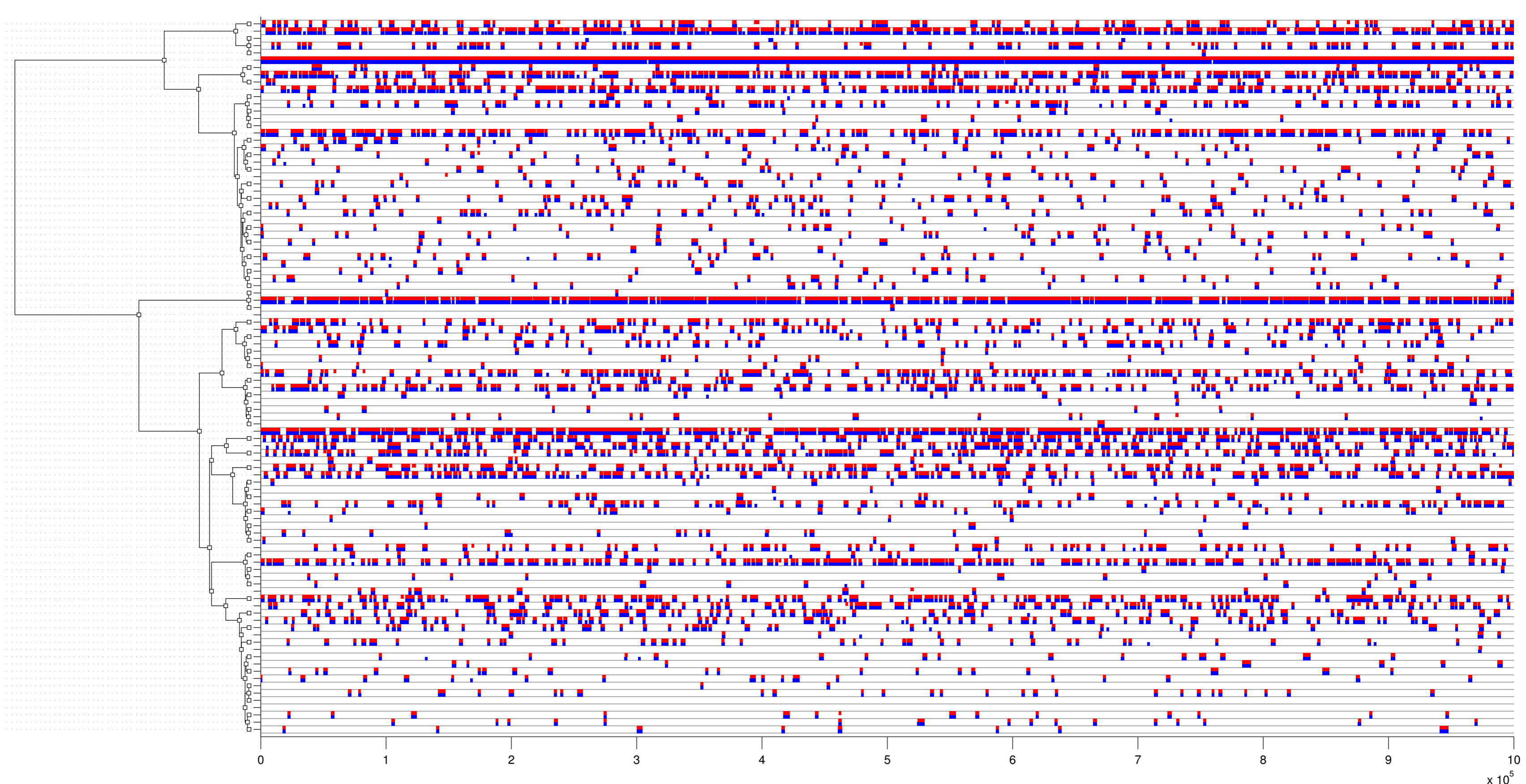

Supplement: S2 Fig — True recombination events are shown in blue and events detected by ClonalFrameML are shown in red. (PDF) [file pcbi.1004041.s002.pdf]

**Without normalization of trees**

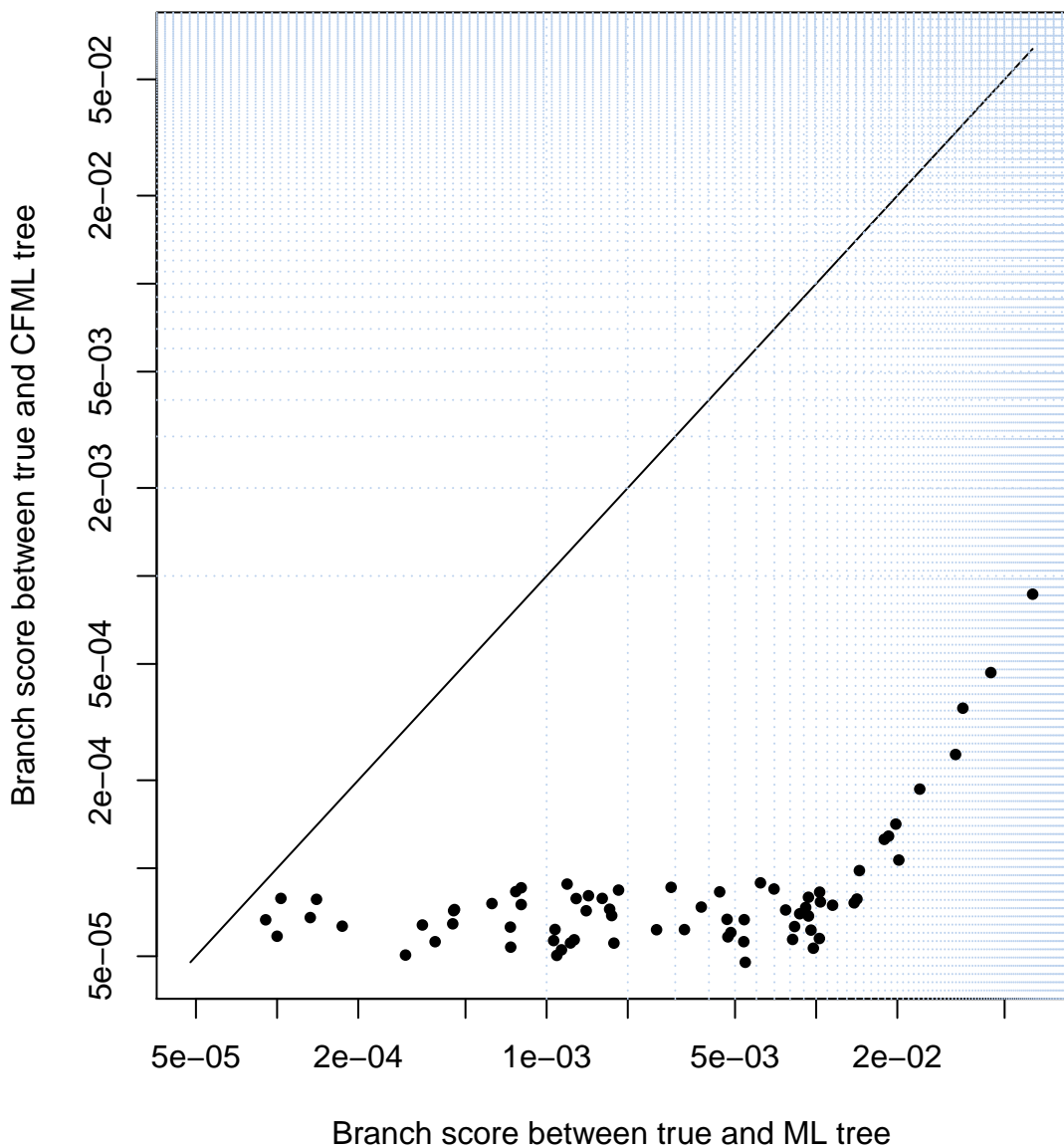

**With normalization of trees**

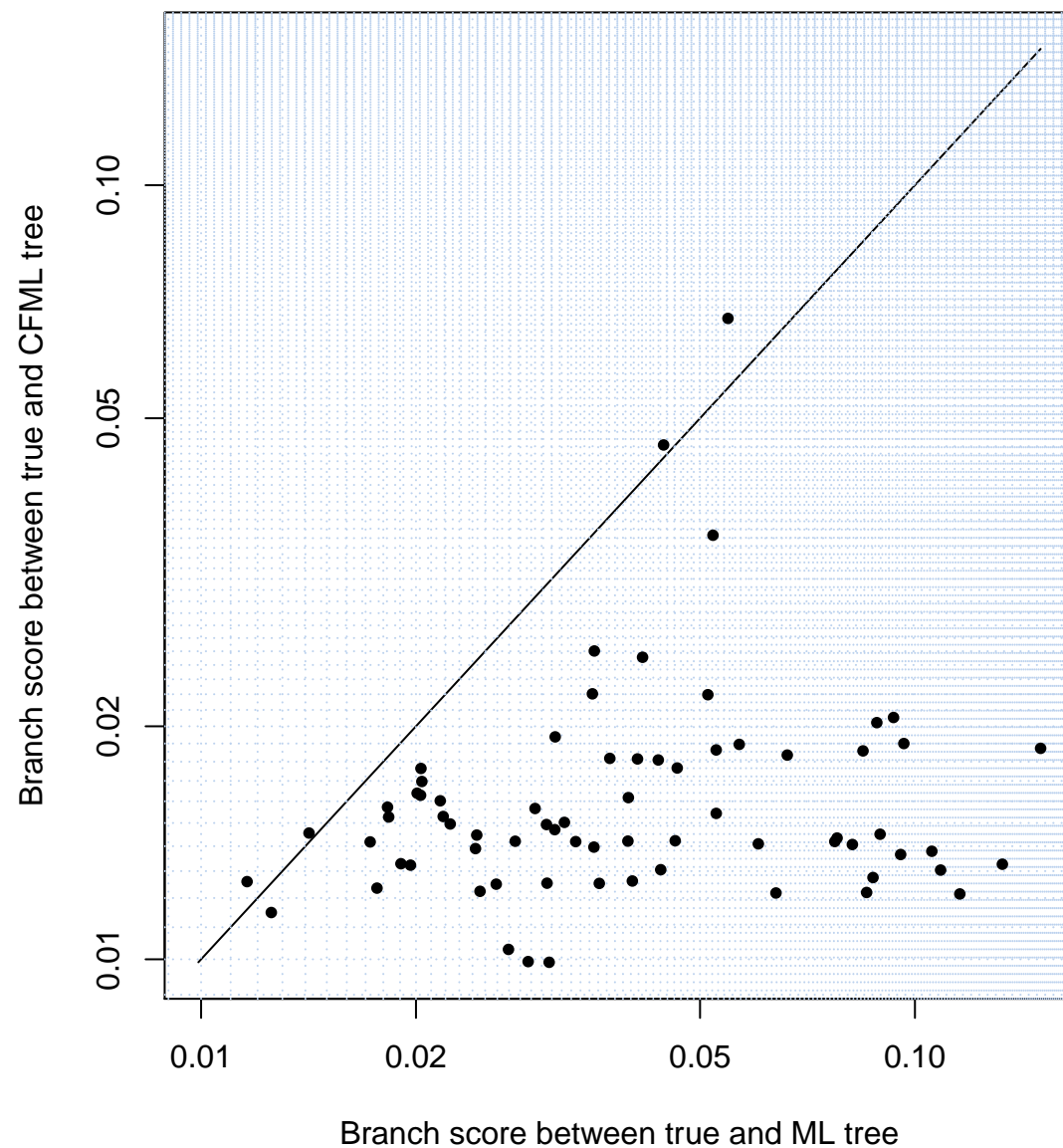

Supplement: S3 Fig — The x-axis shows the branch score between true and ML tree, whereas the y-axis shows the branch score between true and ClonalFrameML tree. In the right panel all trees have been normalized to have a sum of branch lengths equal to one. (PDF) [file pcbi.1004041.s003.pdf]

Distance in ClonalFrameML tree

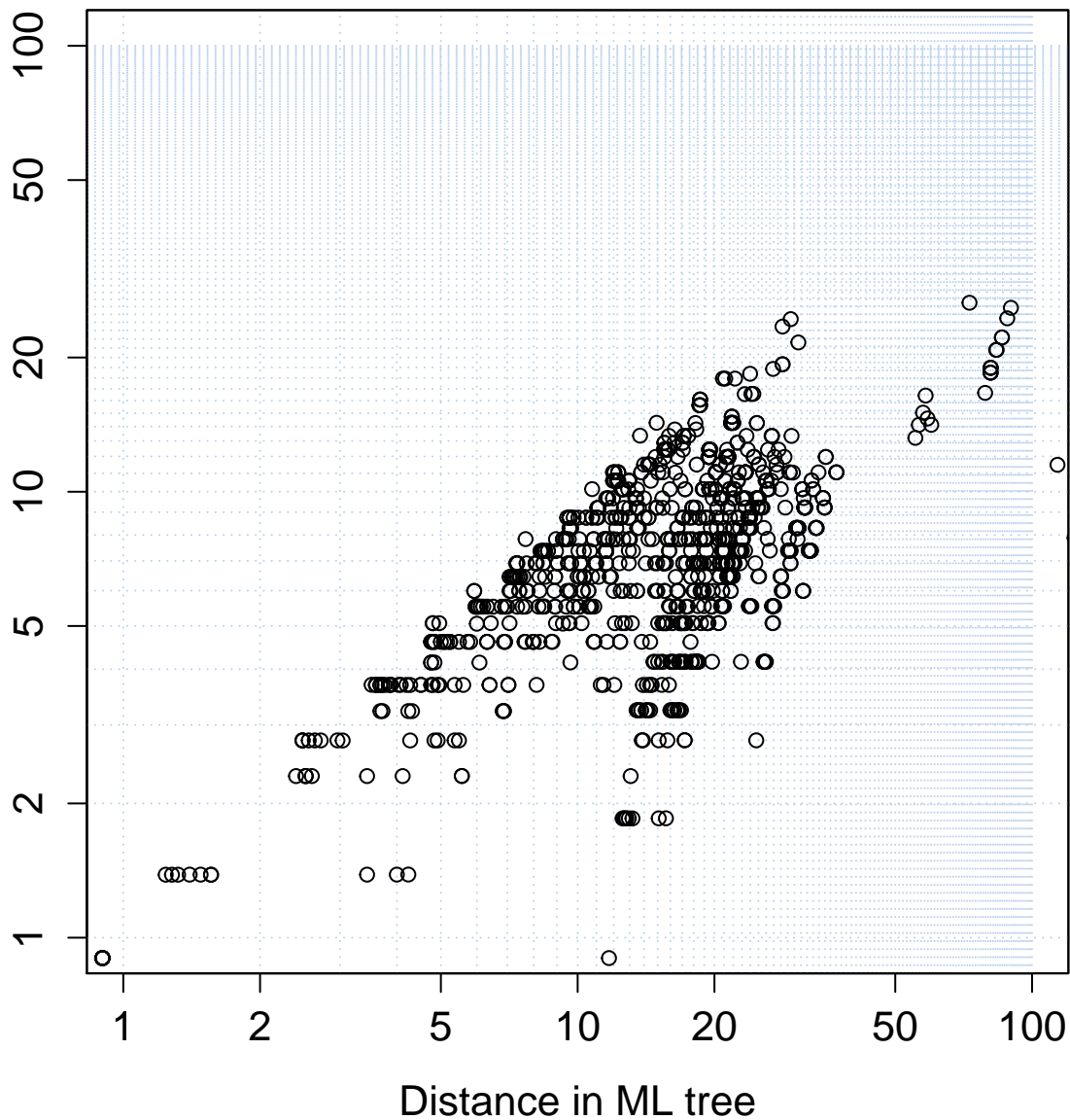

Supplement: S4 Fig — (PDF) [file pcbi.1004041.s004.pdf]
