# Supplementary material for: ClonalFrameML: Efficient Inference of Recombination in Whole Bacterial Genomes
Source: PLoS Comput Biol. 2015 Feb 12;11(2):e1004041. doi: 10.1371/journal.pcbi.1004041 (PMC4326465; doi:10.1371/journal.pcbi.1004041)
Supplement: S5 Fig — The branches of a simplified tree representing S. aureus STs 15, 582, and 20 together with an outgroup representing all other lineages are labelled A-E. A recombination event from ST 20 to ST 582 is labelled R. Below, patterns of genetic diversity are represented for mutations arising on branches A-E in the absence of recombination (clonal sites). Recombined sites show the effect of the recombination event R on patterns of diversity. Mutation events occurring on branch D are imported into lineage A, leading to homoplasy. Mutation events that occurred on branch B are displaced by the recombination event, leading to a spurious pattern resembling mutation on branch E, which we refer to as mirroring. (PDF) [file pcbi.1004041.s005.pdf]

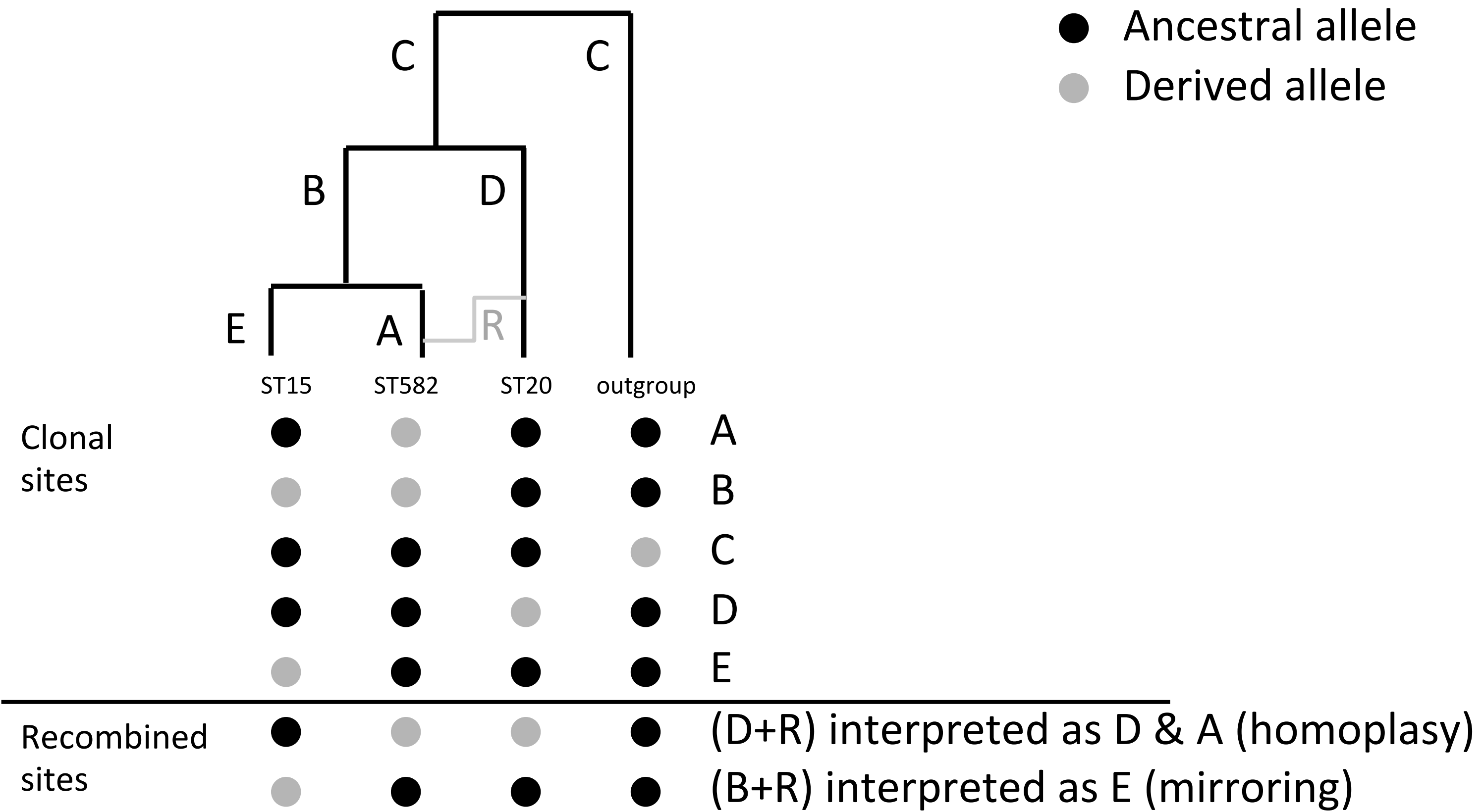
Ancestral allele
Derived allele
C
C
B
D
E
A
R
ST15
ST582
ST20
outgroup
Clonal sites
A
B
C
D
E
Recombined sites
(D+R) interpreted as D & A (homoplasy)
(B+R) interpreted as E (mirroring)
