# Supplementary material for: ClonalFrameML: Efficient Inference of Recombination in Whole Bacterial Genomes
Source: PLoS Comput Biol. 2015 Feb 12;11(2):e1004041. doi: 10.1371/journal.pcbi.1004041 (PMC4326465; doi:10.1371/journal.pcbi.1004041)
Supplement: S1 Text — (PDF) [file pcbi.1004041.s006.pdf]

Introduction

This is an excerpt from the homepage of `ClonalFrameML`, a software package that performs efficient inference of recombination in bacterial genomes. `ClonalFrameML` was created by Xavier Didelot and Daniel Wilson. `ClonalFrameML` can be applied to any type of aligned sequence data, but is especially aimed at analysis of whole genome sequences. It is able to compare hundreds of whole genomes in a matter of hours on a standard Desktop computer. There are three main outputs from a run of `ClonalFrameML`: a phylogeny with branch lengths corrected to account for recombination, an estimation of the key parameters of the recombination process, and a genomic map of where recombination took place for each branch of the phylogeny.

`ClonalFrameML` is a maximum likelihood implementation of the Bayesian software ClonalFrame which was previously described by Didelot and Falush (2007). The recombination model underpinning `ClonalFrameML` is exactly the same as for `ClonalFrame`, but this new implementation is a lot faster, is able to deal with much larger genomic dataset, and does not suffer from MCMC convergence issues. A scientific paper describing `ClonalFrameML` in detail has been submitted.

Download

You can download the C++ source code for `ClonalFrameML` via SVN using the command:

```
svn checkout http://clonalframeml.googlecode.com/svn/trunk/ clonalframeml
```

Alternatively, you can access the files directly in your browser here and save them to a local directory on your computer.

Please note that the code for `ClonalFrameML` is distributed under the terms of the GNU GPL v3 license, for more details see https://www.gnu.org/copyleft/gpl.html

Installation

After downloading the `ClonalFrameML` source code as described above, you can compile it using the following command:

```
cd clonalframeml/src
./make.sh
```

User guide

The user guide for `ClonalFrameML` is available here.

Getting help

If you need assistance using `ClonalFrameML`, you can get in touch by emailing either Xavier Didelot or Daniel Wilson.

User Guide

Please note that this user guide and the software front-end is still under development.

Input

There are two input files needed to run `ClonalFrameML`. The first one is an alignment of the sequences which can be either in fasta format or extended multi fasta (XMFA) format. The second one is a starting tree, which must be in Newick format. There must be as many leaves in this tree as there are sequences in the alignment file, and the names of the leaves must match the names in the headers of the alignment file.

This starting tree can be generated for example using RAxML or PhyML.

Running

The basic command for running `ClonalFrameML` is as follows:

```
ClonalFrameML newick_file seq_file kappa output_prefix [OPTIONS]
```

The `newick_file` and `seq_file` are the two input files described above. The parameter `kappa` specifies the transition/transversion bias, and is an output of the phylogenetic reconstruction software. The `output_prefix` is the prefix used for all output files generated by `ClonalFrameML`.

Output

Running `ClonalFrameML` produces several output files, each of which starts with the `output_prefix` specified in the command line and ending with the following extensions:

ML_sequence.fasta

This file contains the sequence reconstructed by maximum likelihood for all internal nodes of the phylogeny, as well as for all missing data in the input sequences.

position_cross_reference.txt

A vector of comma-separated values indicating the location in the input sequence file of the sites reconstructed in the output `ML_sequence.fasta` file.

em.txt

This file contains the point estimates for R/theta, nu, delta and the branch lengths.

emsim.txt

This file contains the bootstrapped values for the three parameters R/theta, nu and delta.

importation_status.txt

This file contains the list of reconstructed recombination events. There is one line for each event, the first column indicates the branch on which the event was found, and the second and third columns indicate the first and last genomic positions affected by the recombination event.

labelled_tree.newick

This file contains the starting tree with all nodes labelled so that they can be referred to in other files.

Graphical output

To produce a graphical representation of the `ClonalFrameML` output, use the following command:

```
Rscript cfml_results.R output_prefix
```

The result is a PDF file named `output_prefix.pdf`

Example: standard model

Start by downloading the example files from here. Unzip on Mac/Linux as follows:

```
tar -xzvf cfml.tgz
```

The file contains the S. aureus FASTA file (Saureus.fasta), maximum likelihood tree (Saureus.phyML.newick), lists of core and non-core sites (Saureus.core-sites.txt and Saureus.non-core-sites.txt) and some R code (cfml_results_2.R).

Run the standard `ClonalFrameML` analysis as follows. Note! This is a real-world example, and requires around 12 hours of run time.

```
./ClonalFrameML Saureus.phyML.newick Saureus.fasta 4.967695 example.output -em true
-emsim 100 -guess_initial_m true -ignore_user_sites Saureus.non-core-sites.txt
-use_incompatible_sites true -driving_prior_mean
"0.3793988249 0.0037939882 0.3793988249 0.0001199764" -driving_prior_precision
"3.33101e+00 3.33101e+04 3.33101e+00 3.33101e+07" > example.log.txt
```

Note that the transition:transversion ratio of 4.967695 was estimated by PhyML. The option -em true specifies the standard analysis in which recombination parameters are shared by all branches, and the -emsim 100 option requests 100 pseudo-bootstrap replicates. The -guess_initial_m true aims to start the estimation of branch lengths at a reasonable set of values. The positions listed in the file specified by the -ignore_user_sites option lists any site that was not callable or present in all the genomes. These sites are treated as missing data, which is important since uncalled sites can cause artefactual clustering of substitutions. The -use_incompatible_sites option specifies the analysis of homoplasious as well as non-homoplasious sites.

The pseudocounts prior is specified by -driving_prior_mean and -driving_prior_precision corresponding to the parameters of a gamma distribution. They specify the parameters in the following order: R/theta (relative rate of recombination to mutation), 1/delta (inverse mean DNA import length), nu (mean divergence of imported DNA) and mean branch length. The suggested values specify distributions that, on the log scale, are approximately centred on R/theta = 0.1, delta = 1000, nu = 0.1 and the mean branch length in the PhyML tree, with standard deviations of one order of magnitude.

Once the analysis has finished, a figure can be produced with an R script. The R script takes a list of the core sites (the complement of the non-core sites fed into ClonalFrameML):

```
Rscript cfml_results_2.R example.output core-sites.txt
```

This will generate a pdf similar to the figures in the paper.

Example: per-branch model

Run the `ClonalFrameML` analysis in which recombination parameters are estimated per branch as follows . Note! This is a real-world example, and requires around 12 hours of run time.

```
./ClonalFrameML Saureus.phyML.newick Saureus.fasta 4.967695 example2.output
-embranch true -emsim 100 -embranch_dispersion 0.1 -ignore_user_sites
Saureus.non-core-sites.txt -use_incompatible_sites true -driving_prior_mean
"0.3793988249 0.0037939882 0.3793988249 0.0001199764" -driving_prior_precision
"3.33101e+00 3.33101e+04 3.33101e+00 3.33101e+07" -initial_values
"0.141679 350.104 0.0147762" > example2.log.txt
```

This time -embranch is specified instead of -em. The -embranch_dispersion option specifies the constraint on the variability of recombination parameters among branches of the tree. It is on a scale of 0-1, with 0 being the most constrained (least dispersed). The -initial_values of R/theta, delta and nu can be set. It is advisable to run the simpler model first, and use the results to initialize the per-branch model. Again the R code can be used to generate a figure.

Full list of options

The options of `ClonalFrameML` are listed in full below:

```
-fasta_file_list              true or false (default)   Take fasta_file to be a white-space separated file list.
-correct_branch_lengths       true (default) or false   Correct branch lengths using ClonalFrame model.
-excess_divergence_model      true or false (default)   Use the 'excess divergence' model. Mandatory for two sequences.
-ignore_incomplete_sites      true or false (default)   Ignore sites with any ambiguous bases.
-ignore_user_sites            sites_file                Ignore sites listed in whitespace-separated sites_file.
-reconstruct_invariant_sites  true or false (default)   Reconstruct the ancestral states at invariant sites.
-use_incompatible_sites       true or false (default)   Use homoplasious and multiallelic sites to correct branch lengths.
-brent_tolerance              tolerance (default .001)  Set the tolerance of the Brent routine.
-powell_tolerance             tolerance (default .001)  Set the tolerance of the Powell routine.
-joint_branch_param           true or false (default)   Jointly optimize branch lengths and recombination parameters.
-rho_per_branch               true or false (default)   Estimate recombination parameters separately for each branch.
-rho_per_branch_no_lrt        true or false (default)   As above but suppress likelihood ratio test for recombination.
-single_rho_viterbi           true or false (default)   Jointly optimize recombination parameters using Viterbi algorithm.
-single_rho_forward           true or false (default)   Jointly optimize recombination parameters using forward algorithm.
-rescale_no_recombination     true or false (default)   Rescale branch lengths for given sites with no recombination model.
-multithread                  true or false (default)   Enable OpenMP parallel code. Overhead may cancel out gains.
-show_progress                true or false (default)   Output the progress of the maximum likelihood routines.
-compress_reconstructed_sites true (default) or false   Reduce the number of columns in the output FASTA file.
-initial_rho_over_theta       value > 0 (default 0.1)   Initial value of rho/theta used in the search.
-initial_import_divergence    value > 0 (default 0.1)   Initial value of import divergence used in the search.
-initial_mean_import_length   value > 1 (default 500)   Initial value of mean import length used in the search.
-min_branch_length            value > 0 (default 1e-7)  Minimum branch length.
-mcmc_per_branch              true or false (default)   Estimate by MCMC recombination parameters for each branch.
-laplace_approx               true or false (default)   rho_per_branch model with approximation of the joint posterior.
-use_nelder_mead              true or false (default)   Use Nelder-Mead and not Powell method in Laplace approximation.
-viterbi_training             true or false (default)   Estimate parameters by a Viterbi-based hill climbing algorithm.
-driving_prior_mean           4 values (df "0 0 0 0")   Mean of the prior used by Laplace/Viterbi algorithms.
-driving_prior_precision      4 values (df "1 1 1 1")   Precision of the prior used by Laplace/Viterbi algorithms.
-initial_values               3 values/empty (def "")   Initial values used by the Laplace/Viterbi algorithms.
-guess_initial_m              true or false (default)   Initialize M and nu jointly in the Laplace/Viterbi algorithms.
-grid_approx                  value 0/2+  (default 0)   Number of points for a grid approximation (0 = off).
-mcmc                         true or false (default)   Estimate by MCMC recombination parameters for all branches.
-mcmc_infer_branch_lengths    true or false (default)   Estimate by MCMC branch lengths for all branches.
-partial_viterbi              true or false (default)   Estimate parameters by Powell/Nelder-Mead and Viterbi algorithms.
-em                           true or false (default)   Estimate parameters by a Baum-Welch expectation maximization algorithm.
-emsim                        value >= 0  (default 0)   Number of simulations to estimate uncertainty in the EM algorithm.
-embranch                     true or false (default)   Estimate parameters for each branch using the EM algorithm.
-embranch_dispersion          value > 0 (default .01)   Dispersion in parameters among branches in the -embranch model.
```
