# Supplementary material for: ClonalFrameML: Efficient Inference of Recombination in Whole Bacterial Genomes
Source: PLoS Comput Biol. 2015 Feb 12;11(2):e1004041. doi: 10.1371/journal.pcbi.1004041 (PMC4326465; doi:10.1371/journal.pcbi.1004041)
Supplement: S1 Table — (DOCX) [file pcbi.1004041.s007.docx]

| **Symbol** | **Description** |
| --- | --- |
| *R*/*θ* | Per-site rate of initiation of recombination relative to mutation. |
| *δ* | Mean length of DNA imported by homologous recombination. |
| *ν* | Divergence rate, per site, of DNA imported by homologous recombination. |
| *M_i_* | Expected number of mutations per site on branch *i*. |
| *R*/2 | Recombination initiation rate per site per coalescent time unit (*N_e_g* generations). |
| *ρ/2* | Recombination initiation or termination rate per site per coalescent time unit. Other papers use *ρ* in preference to *R*. Note that *ρ* = 2*R.* |
| *θ*/2 | Mutation rate per site per coalescent time unit. |
| *N_e_g* | One coalescent time unit, i.e. the expected time to the most recent common ancestor of two individuals sampled at random from a population. Equal to the product of *N_e_*, the effective population size, and *g*, the duration of a generation. |
| *r*/*m* | Represents the relative impact of recombination versus mutation on the per-site substitution rate. Equal to (*R*/*θ*) × *δ*× *ν*. |
| *H_j_* | Indicates whether the hidden state at site *j* is *unimported* (*U*), i.e. not affected by recombination, or *imported* (*I*), i.e. affected by recombination, on a branch. |
| *d_jk_* | The distance, in nucleotides, between sites *j* and *k.* |
| *A_j_, D_j_* | The ancestral and descendant nucleotides respectively at site *j*, on a branch. |
| $p_{jk}^{\left(t\right)}$ | The transition probability from nucleotide *j* to *k* in time *t* under the HKY85 substitution model. |
| *T_ijk_* | In the EM algorithm, the expected number of transitions on branch *i* between hidden states *j* and *k*. |
| *E_ijk_* | In the EM algorithm, the expected number of observations on branch *i,* given hidden state *j* = *U* (unimported) or *I* (imported)*,* in which the ancestral and descendant nucleotides are the same (*k* = *S*) or different (*k* = *D*). |
| *α_i_*, *β_i_* | Pseudocounts relating prior information regarding parameter *i* = *M*, ν*, δ* or *R*/*θ*, equivalent to a gamma prior distribution on *M*, *ν*, 1/*δ* and *R*/*θ* respectively with scale parameter *α_i_* and rate parameter *β_i_*. |
